# Surgical management of rare benign tumors of the sternum

**DOI:** 10.15406/mojcr.2021.11.00389

**Published:** 2021-06-24

**Authors:** Andrei I Gritsiuta, Alexander Bracken, Patrick Downs, Jorge Lara-Gutierrez, Karisa Beebe, Alexei A Pechetov, Roman V Petrov

**Affiliations:** 1 Department of Surgical Services, University of Pittsburgh Medical Center, USA; 2 Department of Thoracic Surgery, Vishnevsky National Medical Research Center of Surgery, Russia; 3 Department of Thoracic Medicine and Surgery, Lewis Katz School of Medicine at Temple University, USA

**Keywords:** giant cell tumor, osteoma, sternal resection, chest wall reconstruction

## Abstract

Primary benign tumors of the sternum are an exceedingly rare entity. Surgical techniques regarding intervention for these lesions are not clearly defined in the literature given their scarcity. Operative techniques include en-bloc resection of the tumor, and this has proven to be successful in preventing local recurrence despite benign nature of the lesion. Given the often extensive defect created by the excision, reconstruction is frequently necessary; depending on the size of the defect, either autologous bone grafting or the use of synthetic materials may be indicated. This study serves to present two cases of rare primary benign tumors of the sternum, giant cell tumors and osteoma spongiosum and to summarize the available literature. We present a review of the literature of 17sternal giant cell tumor cases reported so far including our patient and unique case of osteoma spongiosum of the sternum, that discusses their surgical management, as well as reconstructive techniques that provided an excellent clinical result and a lack of recurrence on long term follow-up.

## Introduction

Primary chest wall tumors are a very rare entity, with an incidence of 0.04% among newly diagnosed cancers and 5% of all thoracic neoplasms.^1^ The sternum is an uncommon site of bone tumors, the majority of which are malignant (ex. osteosarcomas and chondrosarcomas). Benign lesions represent less than 1% of all primary bone tumors, and sternal neoplasms should be considered malignant until proven otherwise.^2^ The most common types of primary benign sternal tumors are chondromas, bone cysts, and hemangiomas. In this study, we present our experience in the surgical treatment of two extremely rare sternal tumors: giant cell tumors and osteoma spongiosum.

## Patients and methods

The medical records of two adult patients with primary chest wall tumors whounderwent resection of the sternum with anterior chest wall reconstruction from October 2015 to May 2020 were retrospectively reviewed. A systematic analysis of electronic databases was performed including the MEDLINE, PubMed, CINAHL, EMBASE and OVID. A total of 11 appropriate full-text case reports and case series in English language were identified through database and reference searches from 1982 to 2021 for giant cell tumor of the sternum with total number of patients of 16 (Table 1). No sternal solitary osteoma cases have been published in the English literature.

### Case 1: Giant cell tumor

**Figure 1:**
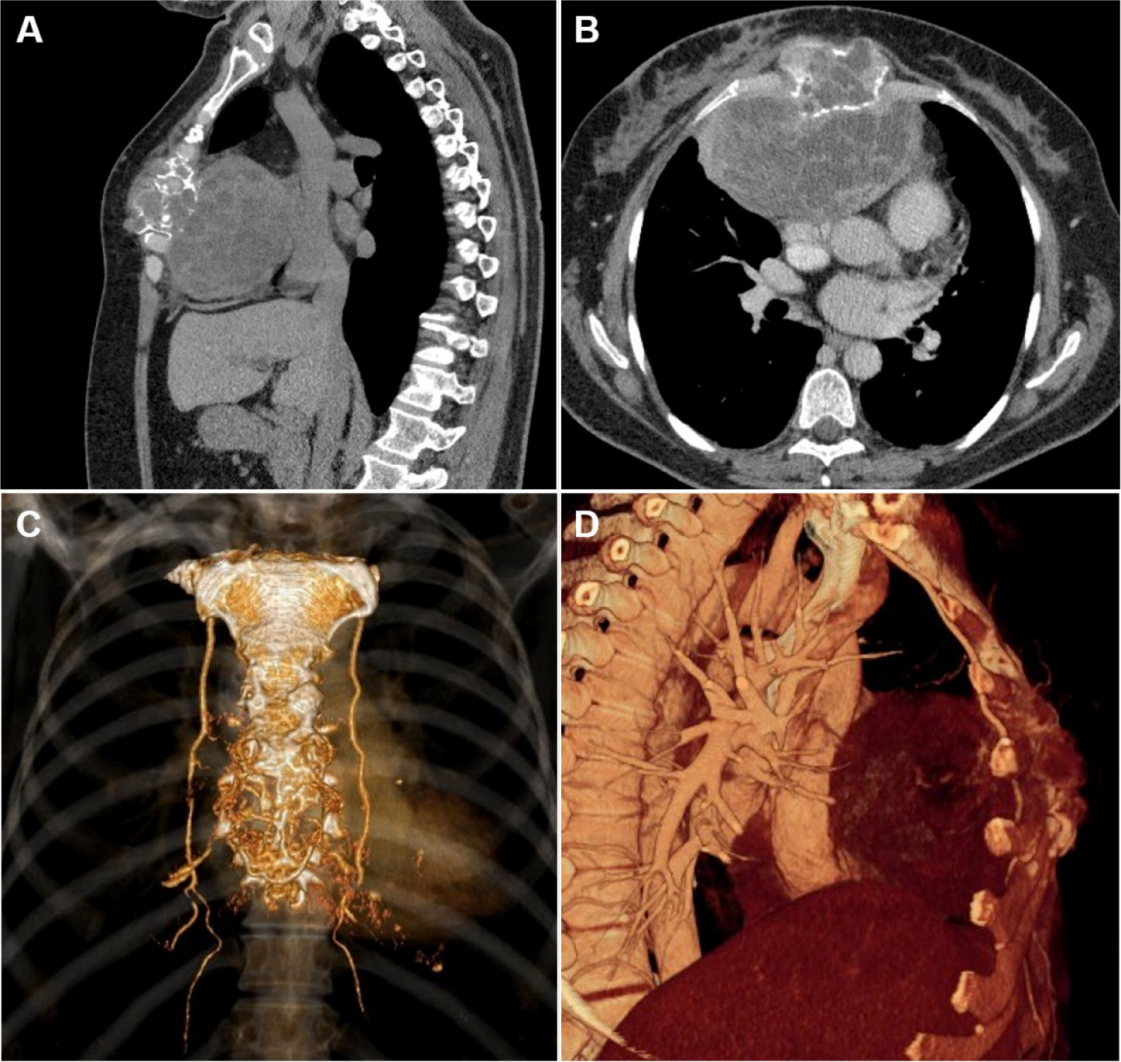
(A) Sagittal and (B) axial chest CT images with (C, D) 3-D reconstruction showing large soft-tissue, osteolytic, heterogeneous sternal mass 12.4cmx9.7cmx8.8cm, with direct extension to the anterior mediastinum, supplied by bilateral internal mammary arteries.

A 50-year-old female with progressive swelling of the anterior chest wall presented with exertional dyspnea, a dry cough, and persistent retrosternal chest pain developing over the course of a year. Physical examination revealed a round, fixed, non-tender 8×10cm dense mass at the mid-lower aspect of the sternum. The overlying skin was not involved. Laboratory values were normal. A chest computed tomography (CT) revealed a 12.4×9.7 ×8.8cm heterogeneous sternal tumor with anterior mediastinal extension. The mass adhered to the mediastinal vascular structures and pericardium, displacing the heart posteriorly and to the left without signs of invasion (Figure 1). Fine-needle aspiration (FNA) cytology was inconclusive. An incisional biopsy established a pathological diagnosis of a sternal giant cell tumor.

**Figure 2:**
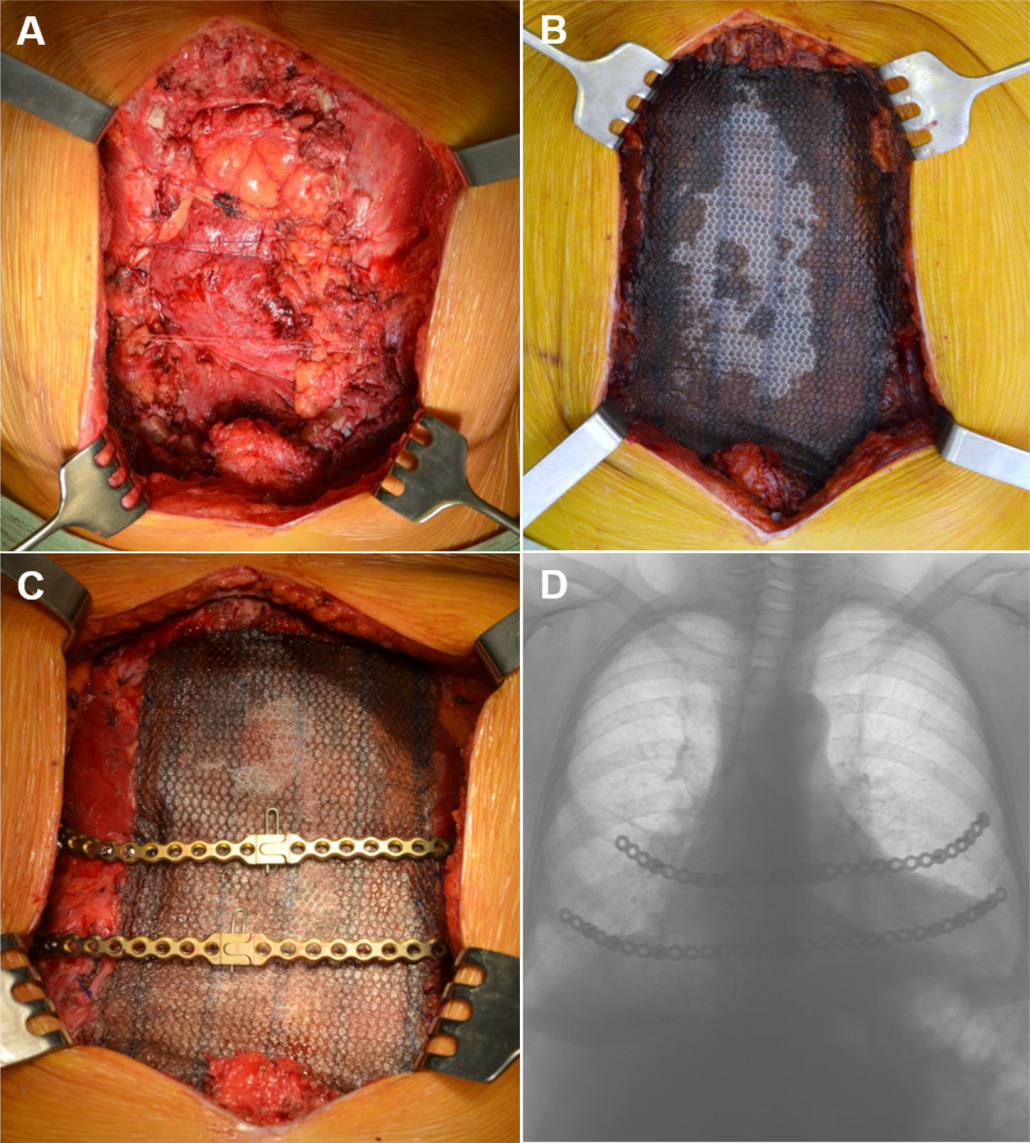
(A) Surgical defect after en bloc subtotal sternectomy for GTC tumor of the sternum. The pleural cavities are closed by approximation of ribs with provisional sutures between the ribs; (B) Application of Prolene mesh for bridging of the defect; (C) Fixation of the titanium plates to the fifth and sixth ribs stumps; (D) Chest X-ray one month postoperatively.

The surgical procedure for management of this lesion began with a midline skin incision over the sternum. Involved cartilages of the 3rd–6th ribs were exposed and excised with wide margin of 2cm from the gross tumor. The sternal resection was performed to macroscopically intact bone tissue (the manubrium was not involved). Internal mammary arteries were identified and bilaterally ligated due to tumor invasion. Wedge resection of the right middle lobe was required due to adherence to the tumor. The resulting defect of the anterior chest wall (10×15cm in size) was covered with a Prolene mesh (Ethicon, Somerville, NJ, USA) (Figure 2 A, B). Stability of the rib cage was achieved by implantation of two titanium plates (Matrix Rib Fixation System, DePuySynthes CMF, West Chester, PA, USA), conformed to the curvature of the chest wall defect and secured to the fifth and sixth ribs (Figure 2C). The prosthesis was covered by bilateral advancement pectoralis major muscle flaps.

**Figure 3:**
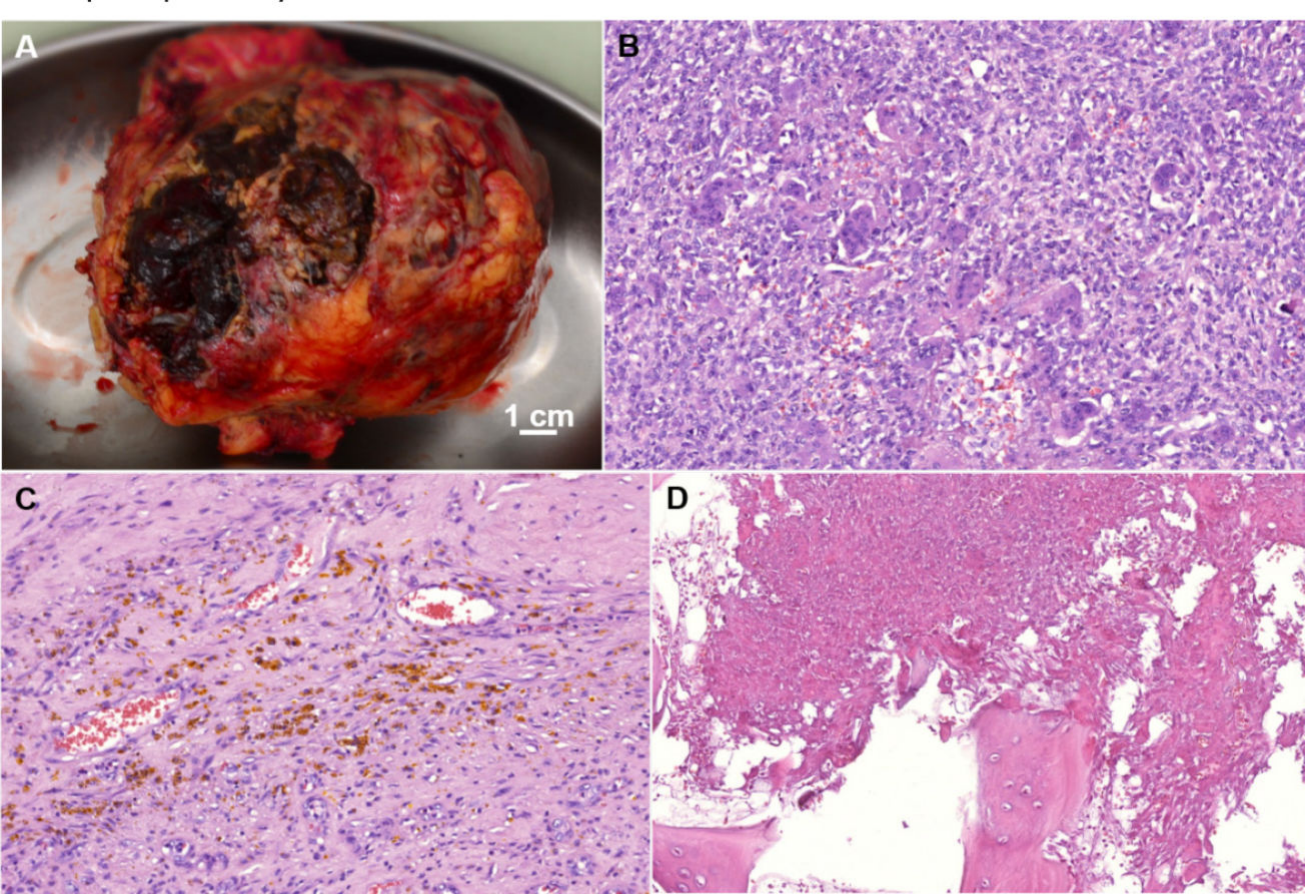
(A) Macroscopic examination of resected GCT; (B-D) Hematoxylin and eosin (H&E)-stained sections of GCT: (B) Giant osteoclast-like multinucleated cells within a background of spindle cell component, 200X; (C) Focal fresh and old hemorrhages, 200X; (D) Tumor destruction of the bone interstitial lamellae, indicating a local aggressive growth of GCT, 200X.

The specimen was a 12cm well circumscribed tumor (Figure 3A), consistent with the preoperative diagnosis of giant cell tumor. Postoperatively, the patient began ambulating on the 2nd day and was discharged home on postoperative day10 (Figure 2D). The patient remains disease free after 6years of follow-up.

### Case 2: Osteoma spongiosum

**Figure 4:**
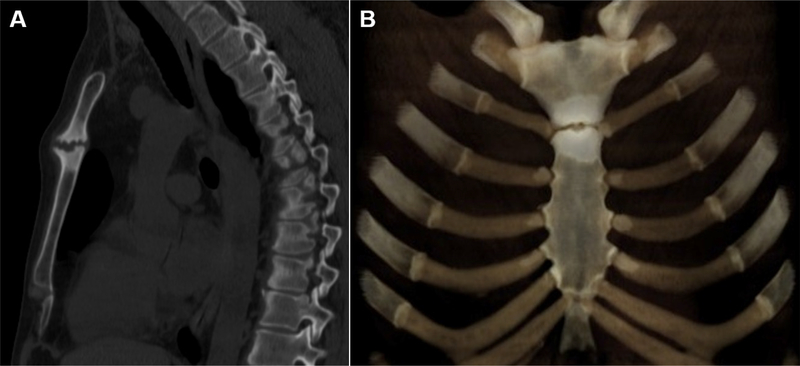
(A) Sagittal CT image (bone window) with (B) 3-D reconstruction demonstrates poorly-defined osteolytic lesion at the sternomanubrial junction with prominent diffused dense sclerosis of the adjacent sternum.

A 42-year-old male presented with uncontrolled, severe, chronic pain in the midsternal region aggravated by minimal activity. Physical examination revealed a 3×4cm area of tenderness and swelling of the upper sternum. Chest CT revealed a cartilaginous mass at the sternomanubrial junction with a widened joint gap, subchondral bone plate with erosive and sclerotic changes, and prominent hyperostosis (Figure 4). Although radiographic changes were read as a chronic inflammatory process, the biopsy revealed concern for a malignant lesion with a differential diagnosis of hemangioendothelioma, osteoid osteoma, osteoblastoma, and well-differentiated osteosarcoma of the sternum.

**Figure 5:**
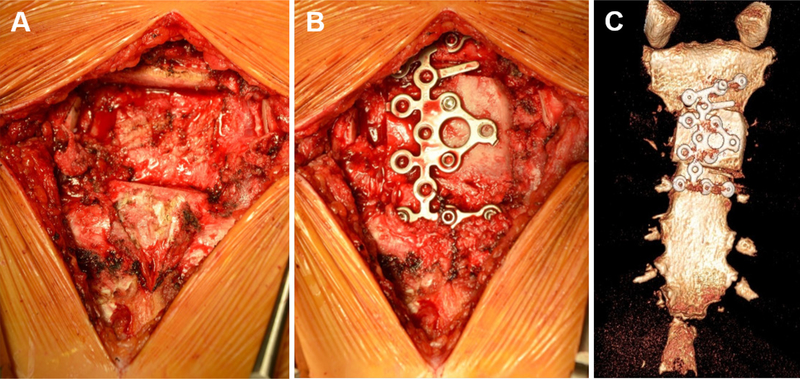
(A, B) Intraoperative images: (A) Partial sternectomy was done following reconstruction with (B) iliac crest autologous bone graft fixed with titanium plate; (C) Sternum CT with 3-D reconstruction one month postoperatively, anterior (pectoral) view.

Planned surgical resection included extirpation of sternomanubrial junction with adequate 1.5cm margins along with bilateral resection of the second and third costal cartilages (Figure 5A). The resulting sternal defect of 3.5 ×4cm was reconstructed with a 4×4cm iliac crest autologous bone graft. Sternal osteosynthesis was achieved utilizing a modified titanium calcaneal plate (Locking Compression Plate (LCP) system, 2 mm thickness, 3.5mm locking screws; DePuy Synthes GmbH, Switzerland) fixed to the sternum and bone graft with eight bicortical self-drilling screws of adjusted length (Figure 5B,C).

**Figure 6:**
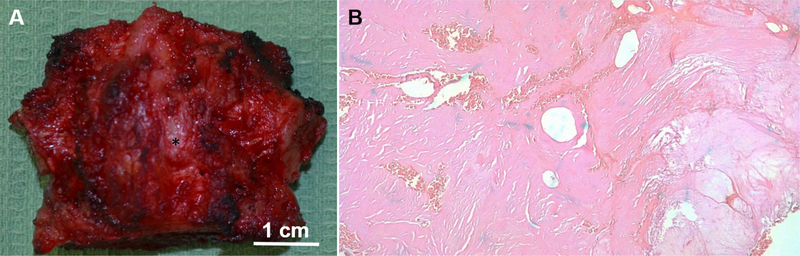
(A) Macroscopic examination of the excised en bloc osteoma of the sternum (asterisk); (B) H&E-stained section of osteoma: discrete large haversian-like fibrovascular channels surrounded by lamellated cancellous-type neoplastic bone with minimal cellularity and foci of woven bone and fibrous tissue, 100X.

The tumor was 1cm in the largest diameter, well circumscribed, consistent with osteoma spongiosum with uninvolved margins (Figure 6). There was no evidence of Gardner’s syndrome. The patient was discharged home on postoperative day 6. He remains well and disease free 5 years postoperatively.

## Discussion

### Giant cell tumor

Giant cell tumor (GCT) of the bone is an uncommon benign neoplasm of mesenchymal origin. GCT consists of proliferation of multinucleated giant cells in the stroma of spindle-shaped mononuclear cells, which are derivatives of monocytes.^3^ The multinucleated cells resemble osteoclasts, but are larger and can contain more than 100 nuclei (average 10 to 50). The ratio of cells that form within the same focus may vary. In addition to round-oval mononuclear cells, often there are sites of proliferation of spindle-shaped mononuclear cells with a relatively large number of mitotic figures (up to 500 in 10 fields of view).^4^

Initially, GCT was described by Cooper in 1818.^5^ The term “Giant cell tumor” for its benign origin was suggested in 1919, differentiated from giant cell sarcoma. GCT most commonly occurs in the third and fourth decades of life, with female predominance (1.5:1 ratio); it does not exceed 10% and 20% of all primary and benign bone tumors, respectively. Despite its benign nature, the tumor can invade surrounding structures, and has a high recurrence rate of up to 50%.^3,6^ Cases of lung metastasis 1 to 10 years after tumor removal have been described.^3,7^ Rarely, less than 2% of malignant GCT, defined as a sarcoma, arise synchronously with GCT, or at the site of a previously resected GCT.^8^

There are numerous classification systems for GCT, but the most useful have been introduced by Campanacci in 1987.^9^ The tumor is classified into three types according to the radiographic appearance: Grade I has a well marginated border and intact cortex; Grade II with a well marginated border without radiopaque rim, the cortex being thin and expanded; Grade III with indistinct borders, cortical destruction, and bulging into the soft tissues. In 1940, Jaffe first classified GCT according to histological characteristics and aggressiveness of the tumor: typical (grade I), aggressive (grade II), and malignant (grade III).^10^ However, an association has never been shown between the histological grading, tumor behavior, local recurrence rate, and prognosis.

Typical (up to 80%) localization of the tumor is in the metaphysis and epiphysis of long tubular bones, particularly around the knee, proximal tibia, and distal radius. Less often, the neoplasm affects the scapula, pelvis, vertebral bodies, ribs and hands.^9^ The sternum is an extremely rare location for GCT. Only 16 cases with a morphologically confirmed diagnosis have been described in the literature (Table 1).^6, 7, 11–22^

Gupta et al.^15^ analyzed histopathological features of 470 GCTs of bone diagnosed over 20 years. In this review, only one patient (0.21%) had a sternal tumor. This rarity was confirmed in a large series of 568 patients with GCT of the bone at the Mayo Clinic, where a 0.3% incidence was found.^22^

According to the literature, the average age of patients with GCT of the sternum is 45 years old (range 28 – 74), which is slightly higher than in patients with GCT of other locations. A differential diagnosis includes more frequent benign (chondroma, fibrous dysplasia, chondroblastoma, aneurysmal bone cyst) and malignant (chondrosarcoma, osteosarcoma, myeloma, malignant lymphoma, desmoid tumor, Ewing sarcoma, metastatic carcinoma) neoplasms of the sternum.7 Osteoclast-like cells are not pathognomonic for GCT, as they can be determined in various inflammatory diseases, benign and malignant bone tumors. In the case of a sternal neoplasm, brown tumors of hyperparathyroidism should be excluded by assessing serum parathyroid hormone and calcium levels.^23^

Fine-needle aspiration is insufficient for the diagnosis of chest wall tumors,and incisional biopsy is required for the accurate morphologic diagnosis of GCT.^14,17,20,21^ The immunohistochemical stains for vimentin, S100, pancytokeratin, desmins, and smooth muscle antigen can be useful in uncertain cases.^11^

Surgical resection remains the only radical treatment option for sternal GCT despite successful bisphosphonate therapy and radiotherapy in selected patients with GCT of the bone.^24,25^ Of note, radiation has no role in the treatment of completely resected tumors.^11^ To date, there are no generally accepted standards of surgical resection of GCT, and recommendations vary from simple curettage to extensive resection.

The extent of resection depends on the tumor location, and subtotal sternectomy is the most common procedure.^6,7,11,13,17,21^ Futani et al.^14^ reported extended curettage of the tumor followed by filling of the defect with polymethyl methacrylate (PMMA) with no recurrence observed 7 years after surgery. Similarly, Segawa et al.^19^ completed the chest wall reconstruction after curettage and cement usage with polypropylene mesh and a titanium mesh plate. This method seems to facilitate recovery, however the curettage has a higher tumor recurrence rate (up to 50%).^26^ PMMA usage was associated with a recurrence rate of 13%, which is still inappropriately high, and this method is not a treatment of choice in sternal localization of the tumor despite its benign origin. Many surgeons prefer methyl methacrylate (MMA) prosthesis for the anterior chest wall reconstruction; this provides mediastinal protection, elimination of flail chest and paradoxical respiration.^6,11,12,17,21^ Some authors used different types of mesh or a polyester patch for the same purpose.^6,11,21^ Engel et al.^7^ reported on the use of a biomaterial mesh only reconstruction without any respiratory complications. Faria et al.^13^ reported successful chest wall reconstruction with fascia lata after GCT resection. In our case, we used titanium plates, achieving the desired supportive function.

Despite the fact that a giant cell tumor of the sternum is an extremely rare disease, this diagnosis should be in the differential diagnostic algorithm for any neoplasm of the sternum and anterior mediastinum.

### Osteoma spongiosum

Osteoma (osteomata) is a slow-growing, benign, osteoblastic bone tumor that most commonly occurs in the third and fourth decades of life with male predominance (3:2 ratio).^27^ Histologically, the neoplasm can be classified into three different types: compact (eburnated), spongy (mature), and mixed.^28^

The entire separate clinical entity is osteoid osteoma (OO), which accounts for approximately 14% of all benign bone tumors, second only to osteochondroma and non-ossifying fibroma. OO most commonly occurs in the first two decades of life with a male predominance (3:1 ratio).^29^ This type of tumor was first described by Jaffe in 1935 as a small, benign osteoblastic tumor measuring 0.5–2cm in diameter. Morphologically similar lesions larger than 2cm are classified as osteoblastomas. Distinctive radiographic characteristics of OO are a central radiolucency (the nidus), usually less than 1cm, with a perifocal sclerotic zone or cortical thickening, while osteomas exhibit as a radiodense mass.^29^ Histologically, the central nidus is composed of intersecting trabeculae of osteoid and woven bone, surrounded by rows of activated osteoblasts alternating with osteoclasts within a dilated vascular fibrous stroma.^30^

Lamellar cortical-type bone, resembling normal bone structure, is diagnostic for osteomas. Spongy osteomas are defined as well-differentiated, mature, cancellous bone with intertrabecular hematopoietic bone marrow and minimal osteoblastic-osteoclastic activity. Osteomas are the most common benign lesion of the skull, most frequently located in the paranasal sinuses, while extracranial localization is extremely rare.^30^ Only 13 cases have been reported in the literature and included tibia, femur, acetabulum, pubis, ilium, ulna, humerus, clavicle and patella.^31^ We conducted a focused literature review of PubMed and MEDLINE databases for similar cases of osteomas in the sternum. We identified only 3 previous reports of sternal OO,^32–34^ and, to the best of our knowledge, no sternal solitary osteoma cases have been published in the English literature.

Surgical resection is the most effective treatment in any type of symptomatic osteoma with a reported success rate of 88 – 100%. En-bloc tumor resection with excision of the surrounding reactive zone reduces the risk of recurrence.^35^ We chose to reconstruct the resultant sternal defect at the sternomanubrial junction with cancellous autologous bone, which is considered to be the “gold standard” bone grafting material, possessing all the necessary basic properties: osteoinduction, osteogenesis and osteoconduction. Autologous bone is histocompatible and nonimmunogenic, thus eliminates the risk of immunoreactions. Dimitriouet al.^36^ reported an estimated morbidity rate of 19.37% in a total of 6449 harvesting cases. Acute pain and sensory disturbances, including hyperesthesia, dysesthesia or diminished sensitivity are the most common complications (up to 62% of patients) at the donor site. Our patient did not experience any of these postoperative events in short- and long-term follow-up.

## Conclusion

This study serves to showcase two rare benign neoplasms of the anterior chest wall that thoracic surgeons may encounter in their practice. Despite the fact that benign chest wall neoplasms are an extremely uncommon entity, GCT and osteoma should be included in the differential diagnosis for any neoplasm of the sternum and anterior mediastinum. With the relative scarcity of literature available to delineate the surgical management of these rare tumors, operative guidelines are not universal. En-bloc resection with wide margins has proven to be a successful approach that has avoided recurrence in long term follow up. Reconstruction is often necessary given the extensive defects created, and should be tackled by either the use of autologous bone grafting or use of synthetic materials. The preferred reconstructive technique is not established. Planning regarding the often complex anterior chest wall reconstruction should be completed utilizing a multidisciplinary approach based on the input from different surgical sub-specialties.

## Figures and Tables

**Table 1:** Surgical treatment of the sternal GCT

Author	Year	Sex	Age	Size (cm)	Location	Resection	Reconstruction
Sundaram et al.^20^	1982	M	55	-	Manubrium	ST sternectomy	-
Bay et al.^12^	1999	F	49	3.9*3.2	Manubrium	ST sternectomy	MMA
Segawa et al.^19^	2004	M	55	3.5*3.0	Body	Curettage	PMMA Polypropylene mesh Titanium mesh plate
Imai et al.^17^	2006	M	45	8.5*4.5*2.5	Body	ST sternectomy	MMA
Futani et al.^14^	2008	F	53	8*4*2.5	Body	Curettage	PMMA
Abate et al.^11^	2009	M	28	6.4*4.3*4.4	Body	ST sternectomy	MMA Prolene mesh
Faria et al.^13^	2010	F	74	10*6*3	Body	ST sternectomy	Fascia lata
Engel et al.^7^	2011	M	32	7,6*5,1*4,7	Manubrium	ST sternectomy	Biomaterial mesh
Traibi et al.^21^	2011	F	34	14*9*8	Body	ST sternectomy	MMA Mersuture mesh
Wang et al.^6^	2015	F	53	6.2*4.9*4.0	Body	ST sternectomy	MMA Polyester Patch
Gao et al.^37^	2018	F	45	6*8	Body	ST sternectomy	Titanium bars Polyester Patch

ST, subtotal; MMA, methyl methacrylate; PMMA, polymethyl methacrylate

## Abbreviations:

CT: Computed tomography
FNA: Fine-needle aspiration
LCP: Locking compression plate
GCT: Giant cell tumor
PMMA: Polymethyl methacrylate
MMA: Methyl methacrylate
OO: Osteoid osteoma
